# Spatial and Molecular Epidemiology of *Giardia intestinalis* Deep in the Amazon, Brazil

**DOI:** 10.1371/journal.pone.0158805

**Published:** 2016-07-08

**Authors:** Beatriz Coronato Nunes, Márcio G. Pavan, Lauren H. Jaeger, Kerla J. L. Monteiro, Samanta C. C. Xavier, Fernando A. Monteiro, Márcio N. Bóia, Filipe A. Carvalho-Costa

**Affiliations:** 1 Oswaldo Cruz Institute, Oswaldo Cruz Foundation, Rio de Janeiro, Brazil; 2 Oswaldo Cruz Foundation, Teresina, Piauí, Brazil; Aga Khan University Hospital Nairobi, KENYA

## Abstract

**Background:**

Current control policies for intestinal parasitosis focuses on soil-transmitted helminths, being ineffective against *Giardia intestinalis*, a highly prevalent protozoon that impacts children’s nutritional status in developing countries. The objective of this study was to explore spatial and molecular epidemiology of *Giardia intestinalis* in children of Amerindian descent in the Brazilian Amazon.

**Methodology/Principal Findings:**

A cross sectional survey was performed in the Brazilian Amazon with 433 children aged 1 to 14 years. Fecal samples were processed through parasitological techniques and molecular characterization. Prevalence of *G*. *intestinalis* infection was 16.9% (73/433), reaching 22.2% (35/158) among children aged 2–5 years, and a wide distribution throughout the city with some hot spots. Positivity-rate was similar among children living in distinct socioeconomic strata (48/280 [17.1%] and 19/116 [16.4%] below and above the poverty line, respectively). Sequencing of the *β-giardin* gene revealed 52.2% (n = 12) of assemblage A and 47.8% (n = 11) of assemblage B with high haplotype diversity for the latter. The isolates clustered into two well-supported *G*. *intestinalis* clades. A total of 38 haplotypes were obtained, with the following subassemblages distribution: 5.3% (n = 2) AII, 26.3% (n = 10) AIII, 7.9% (n = 3) BIII, and 60.5% (n = 23) new B genotypes not previously described.

**Conclusions/Significance:**

*Giardia intestinalis* infection presents a high prevalence rate among Amerindian descended children living in Santa Isabel do Rio Negro/Amazon. The wide distribution observed in a small city suggests the presence of multiple sources of infection, which could be related to environmental contamination with feces, possibly of human and animal origin, highlighting the need of improving sanitation, safe water supply and access to diagnosis and adequate treatment of infections.

## Introduction

Among the intestinal parasites, *Giardia intestinalis* stands out for its high frequency in different socioenvironmental scenarios and its prevalence in both developed and developing countries [1–5]. *G*. *intestinalis* presents high levels of genetic diversity, which have been classified into eight assemblages (A-H). Parasites isolated from humans belong to the globally distributed assemblages A and B, which also have other animals as hosts, being potentially zoonotic [6]. Genotypes C and D have been described in domestic and wild canines, genotype E in domestic ruminants and pigs, F in cats, G in mice and rats and H in seals [7].

Infections with *G*. *intestinalis* occur after the ingestion of cysts in contaminated water, directly from person to person by fecal-oral contamination or, occasionally, from food [7]. Low-income populations residing in environments with poor household sanitation level and without safe water supply are more vulnerable to water and excreta-related diseases. Contaminated central water supplies can be the source of community-wide outbreaks or spreading of *G*. *intestinalis* [4]. Giardiasis prevalence ranges to 20–30% in developing countries and 2–7% in developed countries, being, in the latter, frequently related with day care center disease and public pools outbreaks, and also to travel-associated diarrhea [4,8].

Although *G*. *intestinalis* is an important cause of diarrhea, most infections have chronic and asymptomatic character [9]. The pathogenicity of *G*. *intestinalis* includes apoptosis of enterocytes, epithelial cell damage, and consequent malabsorption [10]. Importantly, *G*. *intestinalis* infection has been shown to impact the nutritional status of children, with the potential of seriously compromising their physical development [11–15].

While control policies for intestinal parasitoses have been successful against soil-transmitted helminths, these same policies are ineffective against protozoan parasites as the treatment for the diseases they cause requires different drugs and more complex ministration schedules [16–18]. Here we assessed the prevalence, spatial distribution and molecular epidemiology of *G*. *intestinalis* infection in children of Amerindian descent that live in a remote municipality in the Brazilian Amazon.

## Materials and Methods

**Fig 1:**
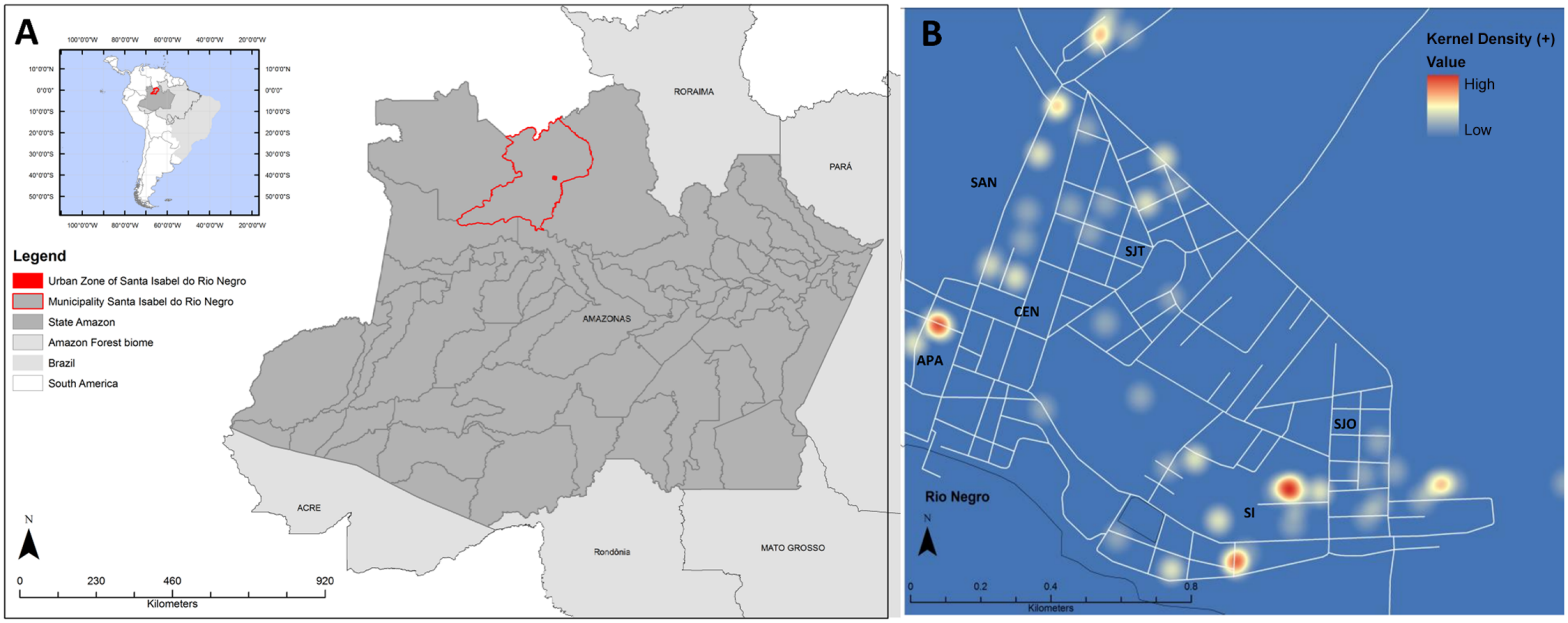
A: Map of the study area in the Brazilian Amazon. B: Hotspot map of *Giardia intestinalis* cases in Santa Isabel do Rio Negro-AM, Brazil, in 2011, generated using the kernel method. Districts: APA- Aparecida; SAN- Santana; CEN- Centro; SJT- São Judas Tadeu; SI- Santa Inês; SJO- São José Operário. The maps were made with data provided by OpenStreetMap available under the Open Database License (https://www.openstreetmap.org/copyright).

This study was a cross-sectional survey performed in 433 children from Santa Isabel do Rio Negro in 2011 (Fig 1A). This small city in Brazilian Amazon was occupied mainly by Amerindians, descendent from the Tukano and Aruak speaking societies. Although the overall population of this area was approximately 18,000 people, this study was conducted in the urban area, comprised of approximately 5,000 inhabitants, distributed among six districts: Aparecida (APA), Centro (CEN), Santa Inês (SI), São José Operário (SJO), São Judas Tadeu (SJT), and Santana (SAN). All children included in our study were at maximum 14 years old. None of them presented with diarrhea during the study. Containers without preservatives were distributed for stool samples collection, and parasitological tests were performed using ether sedimentation technique [19]

Georeferencing was performed with a Global Position System in the SAD-69 geodetic datum. Spatial data were analyzed in a GIS platform using ArcGis 9.3^®^ software (Environmental Systems Research Institute, Redlands, CA-USA). Maps were generated using the kernel density estimation method, and only first order effects were evaluated. The maps were made using data provided by OpenStreetMap^®^ available under the Open Database License (https://www.openstreetmap.org/copyright).

DNA was extracted, in a field laboratory, only from parasitologicaly confirmed *G*. *intestinalis*-positive stool samples using the ZR Fungal/Bacterial DNA kit (ZymoResearch, Irvine-USA). For the amplification of the 753-bp *β-giardin* (*βG*) gene fragment we utilized the G7-G759 primers, as described by Cacciò et al. [20]. Products were purified using the Illustra-GFX kit (GE Healthcare, Pittsburgh, PA-USA) and sequenced with the ABI-BigDye Terminator kit (Applied Biosystems, Foster City, CA-USA) using ABI 3730 (Applied Biosystems) automated sequencer. In addition, sequences that presented double peaks were cloned using pGEM^®^ T-Easy (Promega, Madison, WI-USA). Briefly, the inserts were amplified by PCR using the M13 primer and sequenced [21]. We used the Bioedit-7.1 and Mega-6.0 in order to edit and align the sequences.

Bayesian and maximum-likelihood phylogenetic trees based on 657-bp *βG* sequences were inferred with BEAST-1.8 and PhyML-3.0, respectively. The Akaike and Bayesian Information Criteria of jMODELTEST-2 were used to elect Tamura-Nei with four gamma categories as the best-fit evolutionary model for the dataset. Eighteen orthologous sequences representing the diversity of *G*. *intestinalis* (six of the eight known assemblages) were retrieved from GenBank and added to the analyses. Genealogies were reconstructed with Network-4.6 (Fluxus-Engineering, Inc.) using the median-joining method with maximum-parsimony post-processing.

### Ethics Statement

This study was approved by the Evandro Chagas Research Institute Committee for Ethics on Research of FIOCRUZ (0011.0.009.000–3). The parent or legal guardian of all children included in this study provided written informed consent on their behalf.

## Results and Discussion

The prevalence of *G*. *intestinalis* infection was 16.9% (73/433). Infection was more frequent among children aged 2–5 years old and among males (Table 1). In addition, giardiasis was observed in distinct income strata with similar frequencies.

SI presented a significantly higher *G*. *intestinalis* positivity rate than the other districts (Table 1). A similar trend was observed with the kernel analysis that identified infection hotspots in the APA and SI districts (Fig 1B).

**Table 1:** Distribution of *Giardia intestinalis* infection according to sociodemographic characteristics in Santa Isabel do Rio Negro-AM, Brazil, 2011.

Characteristic	Number of *Giardia intestinalis* positive / examined subjects (% positive)	p-value^a^
**Locality**		
Aparecida	7/47 (14.9%)	0.026
Centro	3/20 (15.0%)	
Santana	18/104 (17.3%)	
Santa Ines	25/87 (28.7%)	
São José Operário	11/104 (10.6%)	
São Judas Tadeu	9/71 (12.7%)	
**Sex**		
Female	29/208 (14.0%)	0.157
Male	44/225 (19.6%)	
**Age (years)**		
0–1	10/60 (16.7%)	0.164
2–5	35/158 (22.2%)	
6–11	25/192 (13.0%)	
12–14	3/17 (17.6%)	
Unknown	0/6 (0%)	
**Income per capita per month (USD)** USD 1 = BRL 4		
Below the poverty line (≤ 38.5)	48/280 (17.1%)	0.854
Above the poverty line (> 38.5 and ≤ 330)	19/116 (16.4%)	
Unknown	6/37 (16.2%)	

^a^Fisher exact test.

**Fig 2:**
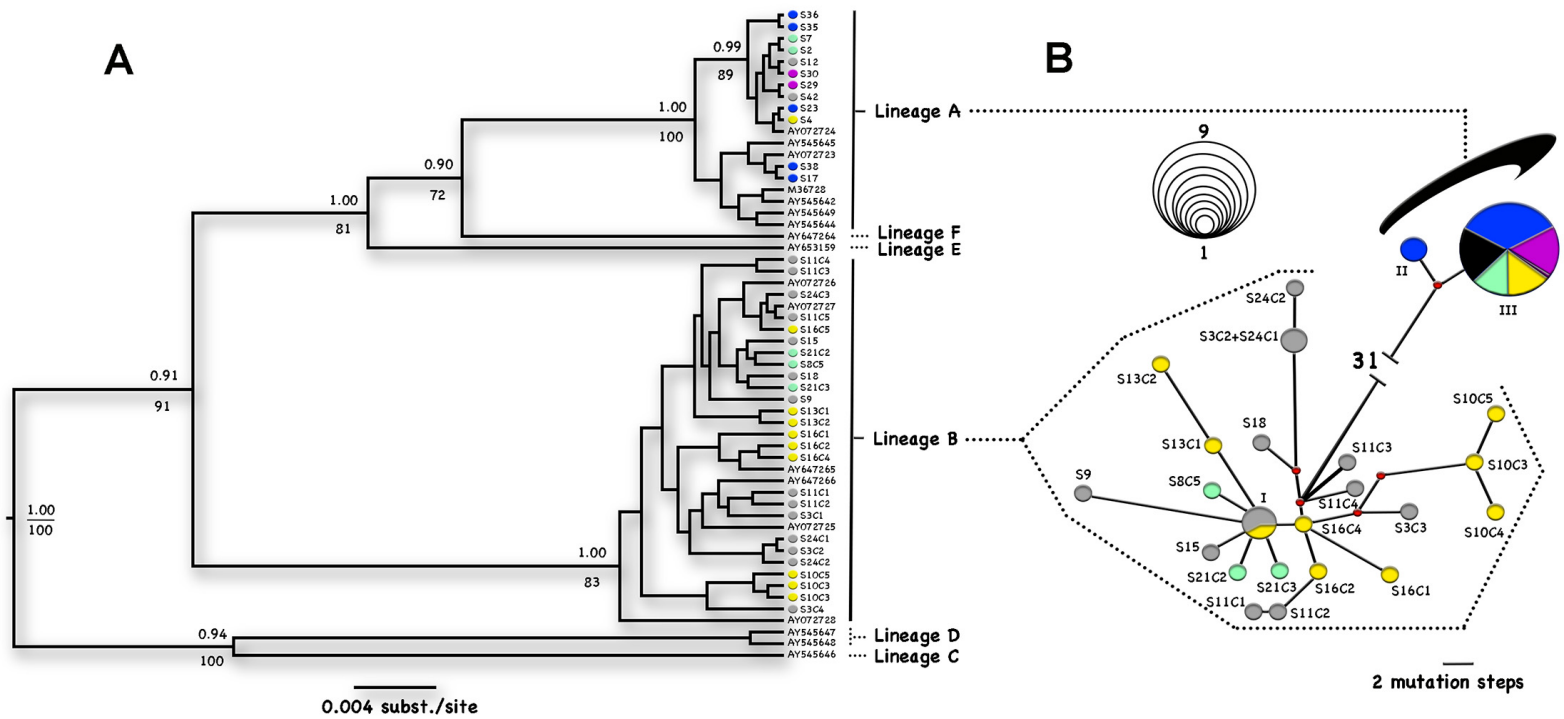
Evolutionary relationships of *Giardia intestinalis*. Each locality is represented by a different color. (A) Phylogenetic consensus tree of the 56 *βG* clone sequences. Posterior probabilities (Bayesian reconstruction) >0.9 and bootstrap values (ML tree) >70 are shown above and below the nodes, respectively. GenBank accession numbers for samples sequenced in other studies are shown in the terminal branches. (B) Median-joining network with maximum-parsimony post-processing. Each circle represents a unique haplotype; nodes represent median vectors. The size of each node is proportional to the number of sequences that shared the same haplotype (see scale) and the branch size is proportional to the number of mutational steps. For improved graphic resolution, the distance between assemblages A and B is not proportional (31 mutational steps). Color key: Blue—APA; Yellow- SAN; Red- CEN; Purple- SJT; Gray- SI; Green- SJO. Sample IDs: I—S11C5, S16C5 and S24C3; II—S17 and S38; and III—S4, S7, S12, S23, S29, S30, S35, S36 and S42. The other IDs concord with Table 2.

Since the extraction was performed in a field laboratory, it was possible to obtain DNA from 50/73 (68.5%) positive stool samples. From these, 23 (46.0%) were good-quality sequences (fragment of 657-bp). The isolates clustered into two well-supported *G*. *intestinalis* clades (Fig 2A). The assemblage frequencies were 52.2% (n = 12) for A and 47.8% (n = 11) for B. While assemblage A sequences obtained did not contain double-peaks, most assemblage B sequences did (n = 7), and were thus cloned. Up to five different haplotypes could be retrieved from a single sample in five clones analyzed.

*G*. *intestinalis* assemblages A and B are widely distributed in the studied region (Fig 2B). Assemblage A exhibited low haplotype diversity, with two haplotypes separated from each other by two mutation steps. One haplotype of assemblage A was observed in five of the six districts, and the other one only in APA. On the other hand, assemblage B exhibited high haplotype diversity (Hd = 0.85), with 22 different haplotypes separated by 1–10 mutation steps. These were present in four districts and only one haplotype was shared between two localities, APA and SI.

A total of 38 haplotypes were obtained, with the following subassemblages distribution: 5.3% (n = 2) AII, 26.3% (n = 10) AIII, 7.9% (n = 3) BIII, and 60.5% (n = 23) new B genotypes not previously described (Table 2). Two distinct epidemiological scenarios were observed. While children living in the same house were infected by the same assemblage, surprisingly children in one house were infected by distinct assemblages (AIII and new B haplotypes).

**Table 2:** Molecular characterization of isolates from Santa Isabel do Rio Negro, Amazonas, Brazil.

Community	Sample ID	Assemblage	Subassemblage	GenBank accession number
Aparecida	S17	A	AII	KU504725
Aparecida	S23	A	AIII	KU504729
Aparecida	S35	A	AIII	KU504735
Aparecida	S36	A	AIII	KU504736
Aparecida	S38	A	AII	KU504737
Centro	S8	B	New	KU504707
Santa Inês	S3C1	B	New	KU504702
Santa Inês	S3C2	B	New	KU504703
Santa Inês	S3C3	B	New	KU504704
Santa Inês	S9	B	New	KU504708
Santa Inês	S11C1	B	New	KU504712
Santa Inês	S11C2	B	New	KU504713
Santa Inês	S11C3	B	New	KU504714
Santa Inês	S11C4	B	New	KU504715
Santa Inês	S11C5	B	BIII	KU504716
Santa Inês	S12	A	AIII	KU504717
Santa Inês	S15	B	New	KU504720
Santa Inês	S18	B	New	KU504726
Santa Inês	S24C1	B	New	KU504730
Santa Inês	S24C2	B	New	KU504731
Santa Inês	S24C3	B	BIII	KU504732
Santa Inês	S42	A	AIII	KU504738
São José Operário	S2	A	AIII	KU504701
São José Operário	S7	A	AIII	KU504706
São José Operário	S21C2	B	New	KU504727
São José Operário	S21C3	B	New	KU504728
São Judas Tadeu	S29	A	AIII	KU504733
São Judas Tadeu	S30	A	AIII	KU504734
Santana	S4	A	AIII	KU504705
Santana	S10C3	B	New	KU504709
Santana	S10C4	B	New	KU504710
Santana	S10C5	B	New	KU504711
Santana	S13C1	B	New	KU504718
Santana	S13C2	B	New	KU504719
Santana	S16C1	B	New	KU504721
Santana	S16C2	B	New	KU504722
Santana	S16C4	B	New	KU504723
Santana	S16C5	B	BIII	KU504724

*G*. *intestinalis* infection was distributed throughout the city, with some hotspots of higher frequency. Interestingly, *G*. *intestinalis* positivity is not associated with income stratum. The wide distribution observed in a small city suggests the presence of multiple sources of infection, which could be related to environmental contamination with feces, possibly of human and animal origin [22]. Many houses do not have access to potable water, being served by two sources: “black water”, which is drawn from the Rio Negro and chlorinated in a plant, and “white water”, which is taken up in wells. The vast majority of homes do not have septic tanks or latrines and the disposal of feces is done directly in the river. This practice may facilitate the spread of different haplotypes of *G*. *intestinalis*. The observed hotspots on the margins of the Rio Negro suggest that people who live closer to the river are at greater risk of becoming infected.

The high genetic divergence between A and B (5.5–6.3%) supports previous proposal for their separation in two taxa, *G*. *intestinalis* and *Giardia enterica* [23, 24]. Assemblage A haplotypes detected in the present study are identical to European strains [20], evidencing their low genetic divergence. In contrast, assemblage B haplotypes were highly diverse, being possible to observe up to five different clones in a single sample. Previous genome sequencing analysis evidenced a 10-fold difference in heterozygosity levels between assemblages A and B [24], but the reasons for such difference are still unknown and deserves further investigation.

The Amazon region is the largest drainage basin in the world and harbors one-fifth of the fresh water reserves on the planet. Paradoxically, the living conditions of many people who inhabit this basin are substandard, favoring the transmission of fecal-oral diseases such as giardiasis. It has been proposed that the routine water treatment practices usually employ concentrations of chlorine able to inactivate only bacterial and viral pathogens, but not *Giardia* cysts [25–26]. Thus, adequate control of giardiasis, particularly in Amazon region requires the improvement of drinking water quality and reduction of environmental contamination with feces [27]. Despite the high prevalence of giardiasis and its health impact worldwide, large-scale interventions—as those implemented for STH control—are lacking in developing countries. In this context, enteric protozoa infections emerge as neglected conditions in the STH control era [18].
